# Current Status and Future Opportunities of Omics Tools in Mycotoxin Research

**DOI:** 10.3390/toxins10110433

**Published:** 2018-10-26

**Authors:** Manal Eshelli, M. Mallique Qader, Ebtihaj J. Jambi, Andrew S. Hursthouse, Mostafa E. Rateb

**Affiliations:** 1 School of Computing, Engineering, & Physical Sciences, University of the West of Scotland, Paisley PA1 2BE, UK; mallique.qader@gmail.com (M.M.Q.); andrew.hursthouse@uws.ac.uk (A.S.H.); 2 Food Science and Technology Department, Faculty of Agriculture, University of Tripoli, Tripoli 13538, Libya; 3 National Institute of Fundamental Studies, Hantana Road, Kandy 20000, Sri Lanka; 4 Biochemistry Department, Faculty of Science, Girls Section, King Abdulaziz University, Jeddah 21551, Saudi Arabia; ejjambi@Kau.edu.sa

**Keywords:** Aflatoxin, genomic, metabolomics, LC-MS/MS, LC-HRMS, ochratoxin, patulin, proteomics, transcriptomics

## Abstract

Mycotoxins are toxic secondary metabolites of low molecular weight produced by filamentous fungi, such as *Aspergillus*, *Fusarium*, and *Penicillium* spp. Mycotoxins are natural contaminants of agricultural commodities and their prevalence may increase due to global warming. Dangerous mycotoxins cause a variety of health problems not only for humans, but also for animals. For instance, they possess carcinogenic, immunosuppressive, hepatotoxic, nephrotoxic, and neurotoxic effects. Hence, various approaches have been used to assess and control mycotoxin contamination. Significant challenges still exist because of the complex heterogeneous nature of food composition. The potential of combined omics approaches such as metabolomics, genomics, transcriptomics, and proteomics would contribute to our understanding about pathogen fungal crosstalk as well as strengthen our ability to identify, isolate, and characterise mycotoxins pre and post-harvest. Multi-omics approaches along with advanced analytical tools and chemometrics provide a complete annotation of such metabolites produced before/during the contamination of crops. We have assessed the merits of these individual and combined omics approaches and their promising applications to mitigate the issue of mycotoxin contamination. The data included in this review focus on aflatoxin, ochratoxin, and patulin and would be useful as benchmark information for future research.

## 1. Introduction

Mycotoxin contamination poses a global challenge for society due to their presence in a wide range of crops [1,2,3,4]. Several reports have described mycotoxicoses outbreaks as a result of mycotoxin contamination in different parts of the world, especially in Africa, America, and Asia [5,6,7,8,9,10,11]. Therefore, maintaining a safe supply of food/feed for human and animal consumption is a critical issue. Mycotoxins are toxic secondary metabolites of low molecular weight produced by filamentous fungi [12]. Aflatoxins, ochratoxins, and patulin are polyketide-derived mycotoxins that are commonly found in crops, leguminous plants, and animal products [13,14]. More specifically, Aflatoxins and ochratoxins belong to the coumarin-type polyketides while patulin belongs to the lactone-type polyketides. Aflatoxins are produced by many species of *Aspergilli* [1,10,13,15,16,17] whereas ochratoxins and patulin can be produced by different genera of *Aspergillus* and *Penicillium* [13,18,19] (see Table 1). All of these fungi can grow on specific crops under favorable conditions of temperature and humidity, and generate mycotoxins before, during, and after harvesting, handling, and shipment [20,21,22]. Aflatoxins are the most widespread group of mycotoxins that are primarily found in cereals, oilseeds [10,15], tree nuts, spices, and milk and dairy products [1,17]. Ochratoxin is another common mycotoxin produced during the storage of different crops such as cereal crops, nuts and dried fruits [6,12,22] whereas patulin is common in fruit and vegetable-based products, mostly apples [23,24]. Aflatoxin B_1_ (AFB_1_) is the most potent natural product that is classified as group A carcinogen. Ochratoxin A (OTA) is classified as group 2B-possible human carcinogen while there is no adequate information related for the carcinogenicity of patulin in experimental animals conducted and no evaluation of the carcinogenicity of patulin to humans. Therefore, patulin is classified as group 3 on the International Agency for Research on Cancer (IARC) classification [10,24,25].

Human and animal food/feed supplies contaminated with mycotoxin not only result in health hazards but also causes major economic loss [27]. Therefore, it is necessary that strategies are developed to control pre- and post-harvest mycotoxin contamination in crops. In the last 50 years, scientists globally have investigated mycotoxin biosynthesis and reported the presence of genetic factors, biotic, and abiotic elements that affect mycotoxin production [1,16,28]. While legal regulation has been implemented by many countries for food quality assurance, establishing guidelines to control mycotoxins is still a challenge throughout the world, particularly in developing countries where the balance between sufficient food supply and the quality of food is an issue [29,30]. However, developing effective strategies to control mycotoxin production during pre-harvesting phase requires the implementation of “high-throughput” omic tools which have the potential to provide a better understanding of mycotoxin issue [31].

**Figure 1:**
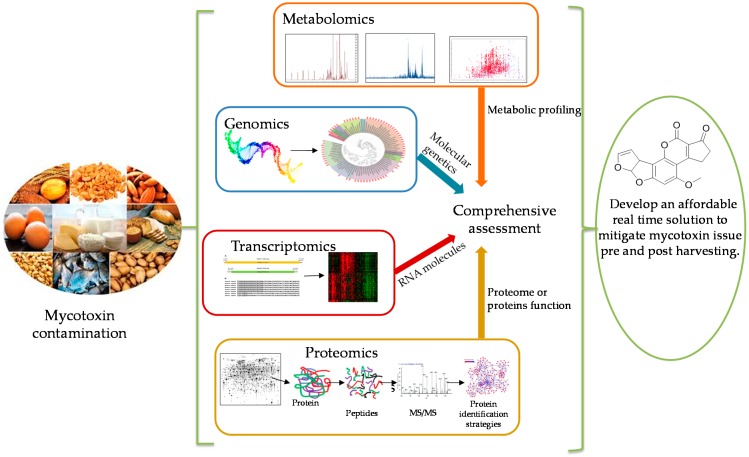
Combining “omics” approaches in mycotoxin research.

Omics tools can be used as a platform to drive hypothesis-based investigations to suggest strategies to control mycotoxin production, for example, using a bio-competitive strategy [32]. Genomics, proteomics, transcriptomics, and metabolomics classify the main disciplines of the wider omics family of technologies (Figure 1). Omics approaches have been rapidly taken up in many fields over the last ten years including food, environmental, medical, molecular, and natural sciences. More than 36,000 research articles related to omics have been published over the last ten years in PubMed that demonstrates the increasing interest exponentially in omics technology in the modern era [33].

Using omics tools in mycotoxin research provides new insights into the genetic constitution of filamentous fungi, information about fungal responses to different ecological factors [14,34] as well as information about mycotoxin biosynthesis under various of environmental conditions [31]. Omics tools can subsequently play a significant role in identifying the microbial strains that can be used against mycotoxigenic strains of *Aspergillus* or *Penicillium* by suppressing their mycotoxin production [35] or identifying the plant constituents that inhibit mycotoxin production [36].

In 2008, Bhatnagar et al. reviewed the potential of omics tools including genomics, proteomics, metabolomics for solving the aflatoxin contamination problem [14]. They indicated that the aim of using omics tools in recent studies is to get a comprehensive assessment of the molecules that make up biological samples such as the cell, tissue or organism. Additionally, assessment of cellular RNA, proteins, primary and secondary metabolites facilitates the study of the fundamental cellular pathways in the host plants and fungi which provides new opportunities to solve food safety problems by interrupting the probability of pre-harvest infection [14,37]. Another recent review has discussed the omics contributions in understanding mycotoxin production under diverse environmental conditions [31]. Although omics tools are less prominent in the practical application, they have already started to yield practical food safety solutions such as array-based biosensors for multiplex mycotoxin analysis [32,38]. However, the challenge for the scientists is to provide reliable data to support the risk assessment of foodborne mycotoxins [4]. In this current review, we highlight these omics approaches and their promising applications to mitigate the issue of mycotoxin contamination.

## 2. Metabolomics Approach

The Metabolomics approach reveals the primary or secondary metabolites that are present in the metabolome of a biological sample under a given set of conditions, which is known as phenotype [39,40]. The metabolome is the collection of all low molecular weight metabolites (small molecules of *MW* ≤1000 Da), which are produced by a living cell during their metabolism, and provides the closest insight to the physiological behaviour of the cell [41]. The metabolic profile of a biological sample shows the metabolites that are depleted or overexpressed in response to different environmental, genetic, pathological, and developmental conditions [41,42,43]. While the analytical technology is advancing rapidly, there are still significant gaps in our knowledge of the biochemical crosstalk between the pathogenic fungi and the host plants/crops. Metabolite profiling or metabolome analysis represents the new tool that facilitates our understanding of fungal cell factories [44,45]. Therefore, metabolites reflect how the cell functions [41,46]. In metabolomics, we search for metabolite differences in the metabolic state of a biological system under investigation condition with the aim to identify metabolites changes under the experimental conditions. This technique could measure the functional phenotypes or fingerprint of biochemical perturbations of the sample before/during the crop invasion by mycotoxigenic fungi, and during the process of contamination by mycotoxins [14,37,47]. Metabolomics studies can be classified into targeted or non-targeted approaches [45,48]. The targeted metabolomics approach is usually used when the scientist has a set of metabolites to measure or specific question to answer. For example, Eshelli et al. used a targeted metabolomics method to investigate the biodegradation of Aflatoxin B_1_ [43]. However, the non-targeted approach is used to identify as many metabolites as possible without any attention to specific metabolites [34]. Therefore, non-targeted metabolomics relies on databases that have been generated globally to capture the information from other metabolomics studies such as METLIN, ChemSpider, and PubChem.

When looking at mycotoxin research, it was reported that the fungal metabolomes change during the fungal growth or the fungal development on the host plant [49] and in the presence of other microorganism or in response to the environment variations [40,50]. Few studies have used these changes in the metabolome to identify the pathogenic fungi such as the classification of *Aspergillus flavus* strains is based on its chemical markers produced through active gene expression [50,51,52]. A recent review by Garcia-Cela et al. indicated that metabolomics is used for identifying and predicting the function of unknown genes by comparison with the metabolic markers caused by a genetic manipulation such as gene deletion or insertion [31]. Falade et al. have investigated the metabolites produced during fungal growth on maize and their correlation with aflatoxin levels. They indicated that metabolites including trehalose, mannitol, and sorbitol are significant for the accumulation of the aflatoxin [49]. Another study used metabolomics tools to detect mycotoxins accumulation in different crops. Aflatoxins accumulation occurs in different concentrations with limit of quantification of AFB_1_ 3.0 µg/kg, AFB_2_ 10.0 µg/kg, AFG_1_ 10.0 µg/kg, AFG_2_ 8.2 µg/kg, AFM_1_ 7.9 µg/kg, and OTA 15.0 µg/kg, and OTB 9.9 µg/kg when the fungal development occur on different substrates [53].

### Analytical Techniques Used in Mycotoxin Metabolomics Studies

Initial trials focused on quantifying a single mycotoxin. Thin layer chromatography was first employed by Scott et al. in 1970 and successfully used to identify eighteen mycotoxins including aflatoxins B_1_, B_2_, G_1_, G_2_, and ochratoxin A using a general solvent system of toluene-ethyl acetate-formic acid or benzene-methanol-acetic acid followed by spraying *p*-anisaldehyde spray reagent [8]. The active fluorescence mycotoxins, AFB_1_ and OTA, were observed as green and blue fluorescence spots under short and long ultraviolet wavelengths, respectively. Subsequently, research moved to the determination of multiple mycotoxins for quantitative and screening purposes. Consequently a range of chromatographic techniques have been used such as high pressure liquid chromatography (HPLC) and gas chromatography (GC) that stand alone or coupled in more advanced instruments as what is referred to as “hyphenated techniques” such as gas chromatography coupled to mass spectrometry (GC-MS), gas chromatography-tandem mass spectrometry (GC-MS/MS), liquid chromatography coupled to mass spectrometry (LC-MS), liquid chromatography-tandem mass spectrometry (LC-MS/MS), and liquid chromatography coupled to nuclear magnetic resonance and mass spectrometry (LC-NMR-MS). These hyphenated systems are currently the most widely applied analytical tools for the detection of mycotoxins [3,41,54,55,56,57,58].

Changing from HPLC to ultra-high pressure liquid chromatography (UHPLC) enhances the ability to detect more metabolites in shorter runtimes. The UHPLC coupled quadrupole-orbital ion trap MS method was used to detect 26 common mycotoxins including aflatoxins B_1_, B_2_, G_1_, and G_2_, ochratoxin A and B in commercially available finished grain or nut products from corn, rice, wheat, almond, peanut, and pistachio [59]. This method showed the potential of hybrid mass analysers for the detection of mycotoxins in food commodities [59]. Rubert et al. used UHPLC-QTOF to investigate plant-pathogen crosstalk and follow the changes in the metabolic fingerprinting, which led to the development of biomonitoring tools for early detection of mycotoxins in wheat [60]. Another study by Oplatowska-Stachowiak et al. used UHPLC-MS/MS to determine the mycotoxin content in dried grain [61]. LC-MS/MS appears to be the most applied technique for the analysis of targeted or known mycotoxin where the analytes are brokedown or fragmented (collision induced fragmentation) and these fragment ions are subsequently detected. Of these systems, triple and quadrupole mass analysers are the most common. Operated under a single reaction monitoring (SRM) mode the triple quadrupole analysers provide high sensitivity and selectivity increasing the possibility of the detection of mycotoxins at the micrograms scale. LC-MS/MS (qQq) can determine, qualitatively and quantitatively, the targeted mycotoxins [62]. While the mass analyser works well in the targeted analysis of mycotoxins, the main limitation of the LC-tandem MS/MS analyser is its ability to detect only targeted or known metabolites not the transformed or modified mycotoxins, unless they are pre-identified [63]. The selectivity and sensitivity of the metabolites depend on the MS analyser. The development of triple quadrupole (qQq) MS analysers played a significant role before the introduction of HR-MS (high resolution) analysers [54]. As qQq mass analysers meet the standard requirements that are necessary for the identification of mycotoxins, they provide cost-effective instrumental access and the capability for MS^n^ determination of toxins [54,64]. Malachova and co-authors were able to optimize and validate a quantitative liquid chromatography-tandem mass spectrometric method. They not only covered all regulated mycotoxins in four model food matrices, but also detected 295 metabolites [65].

LC-HRMS is a convenient analytical tool to approach the tentative identification of targeted as well as untargeted mycotoxins making it ideal for metabolomics study [66]. Considering the advantages of both techniques for mycotoxin determination, the LC-HRMS approach was found to be more appropriate as it has an enhanced resolution compared to LC-MS/MS. The accurate masses obtained from the LC-HRMS provides information on all ions generated and do not depend on ion fragments for identification. Additionally, the ability of its full scan mode allows the identification of targeted and non-targeted compounds [54,62]. Researchers recently focused on method optimisation for untargeted metabolomics such as chemometric analysis that provides a powerful tool to distinguish between different metabolites [60]. Chemometric analysis is the analysis that was utilised to analyse the mathematical and statistical designs to provide the most relevant chemical information by analysing chemical structures and get data concerning biochemical systems [60,67,68]. A recent study indicated that smart chemometrics-assisted analytical strategy that combines with liquid chromatography-full scan-mass spectrometry for multi-mycotoxins analysis in complex cereal samples without sufficient clean-up step [69]. However, with the LC-HRMS, it is possible to design workflow for targeted and routine quantification as well as for untargeted metabolomics and qualitative analysis with the same instrument [64]. The Time-of-Flight (TOF) and Orbitrap HR-MS techniques are the most widely used instruments for the untargeted determination of mycotoxin metabolites [43,64,70] because of their ability to tentatively identify the screened targeted and untargeted compounds, high sensitivity and selectivity, and accurate mass measurement/mass resolution [66]. A recent review by Righetti reviewed recent advances and future challenges in modified mycotoxin analysis, and highlighted why HRMS has become a key instrument in mycotoxin research [64].

Limit of detection (LOD) and limit of quantification (LOQ) are two attributes used to validate the analytical method and to assess the likelihood that mycotoxins are within the regulatory limits. Low LOQ and LOD values are needed for comprehensive and sensitive detection of mycotoxins in samples [54,71]. Thus, it is possible to use these validated methods to enforce regulatory limits on the mycotoxin detection in food commodities. Several targeted and non-target metabolomics for aflatoxin, ochratoxin and patulin detection are highlighted in Table 2 where the available data are presented for LC-MS-based targeted or non-targeted techniques (LC-MS/MS, LC-HRMS) used for the detection of aflatoxins, ochratoxins, and patulin. Apart from LCMS based techniques, GC-MS has also been used in mycotoxin detection. In Table 2, different types of matrices (cereals, seeds, spices, etc.) have been tested. The matrix effects are one of the major concerns in the detection of mycotoxins due to the possibility of masking or overestimating the detection of analytes by co-extracted compounds, which influence the final result. Therefore sample pre-treatment, extraction, and clean-up methods are required, but are not covered specifically in this review. This has been recently reviewed by Malachova and co-authors [62].

## 3. Genomics Approach

A genome consists of all the genetic material contained in a cell of an organism and contains all the necessary information for life. More specifically, it is the complete set of nuclear DNA (coding and noncoding DNA) as well as the genetic material that contain their DNA as in the mitochondria or chloroplast. The genome contains the specific instructions that are necessary for the organism to build and maintain itself [39]. In general, this cell-specific information is encoded in genes, which contains information to code proteins. However, to understand the cellular functions, it is necessary to know the function of all the proteins and the relationship between genes that are expressed (or which proteins are present).

The first complete DNA sequence was obtained in 1992 from the *Saccharomyces cerevisiae*, and in 1995, the bacterial genome of *Haemophilus influenza* was also sequenced [39]. The sequencing of the human genome was completed in 2004, which took around twelve years [91]. The process of (1) characterisation of the structure of the genome of an organism, (2) comparison of sequence genomes with related organisms, and (3) finally, identification of the functions and the interactions of the synthesised proteins or gene is known as genomic analysis [14]. Identifying and interpreting the genomes of a biological sample and characterising their functions that are associated with these genes will give an overall picture of the biological sample. Functional genomics help to understand the interaction between the fungus and its host plant that provides insight about the plant-fungal gene interaction and mycotoxin production [14]. This information assists researchers in developing strategies to control mycotoxin production [4].

The genomics approach can be considered as a pre-harvest application to identify genes that are responsible for the mycotoxin production. Several genomic studies on mycotoxigenic fungi have been carried out so far, especially on aflatoxin producing *A. flavus* [92,93,94,95]. The first genomic analysis of the *A. flavus* was completed by the Food and Feed Safety Research Unit of Southern Regional Research Center, USDA/ARS, under the Expressed Sequence Tags (EST) project and identified more than 7200 unique EST sequences [96]. More recently, the complete genomic analysis was completed by J. Craig Venter Institute, USA using sophisticated and modern bioinformatics techniques where more than 12,000 functional genomes were identified from *A. flavus* [97]. Bioinformatics tools identified that, among them, the coding/encoding protein enzymes are involved in aflatoxin production [98]. Recently, different genomic tools have been used to fulfil the aim of the research, for example, Ion Torrent Personal Genome Machine (PGM), microarray analysis, quantitative reverse transcription-PCR (qRT-PCR) [99,100,101]. Many research studies used different matrices and different types of genetic tools to identify the genes that are responsible for the mycotoxin production and can develop or validate screening methods. Additionally, from the genomic approach, it is possible to identify and differentiate the mycotoxin-producing genes in wild types as well as mutant strains. Table 3 summarises the uses of genomics tools in mycotoxin research.

### Genomics Analysis for Mycotoxin Producing Fungi

*Aspergillus flavus* has played an essential role in the advance understanding of aflatoxigenic genes, biosynthetic pathways, aflatoxin metabolism, the effect of secondary conditions like abiotic conditions on the aflatoxin production, biotic interaction with plants, animals, and humans [105]. Additionally, genomic analysis of other *Aspergillus* spp. and a comparative study of that with the genomes of *A. flavus* allowed for better understanding of the aflatoxins or other mycotoxin producing ability of other *Aspergillus* spp. and their pathogenicity [99]. It is noteworthy that not all *Aspergillus* spp. are toxigenic species. For example, *A. oryzae* has a great economic impact due to its extensive use in food fermentation process in South-East and East Asian countries for the production of soy sauce [95,106]. Morphologically, both *A. flavus* and *A. oryzae* are similar. DNA comparability between *A. flavus* and *A. oryzae* indicates about 98% similarity, and thus, both *A. flavus* and *A. oryzae* contain similar genome size; 36.8 and 36.7 Mb, respectively [95].

Therefore, DNA sequencing of field fungal isolates and their comparison with the gene sequence of aflatoxin-producing *Aspergillus* section *Flavi* would be crucial to identify target genes and control aflatoxin production in crops [107]. Faustinelli et al. have isolated 240 *Aspergillus* strains from peanut seeds during 2014. The genome sequence of all isolates was carried out using Next-Generation Sequencing analysis. They were able to categorise these 240 isolates into nine clades, and among them, three non-aflatoxigenic, five aflatoxigenic *A. flavus* species, and one *A. parasiticus* were identified [108]. Another study used the functional genomics to assess the climate change impact on *A. flavus* and aflatoxin production; the study revealed that global temperature, water availability and rising CO_2_ levels affect the expression of the aflatoxin biosynthetic regulatory gene *aflR* [109].

Recently, the number of studies investigating the gene sequence of ochratoxin A (OTA) producing species to identify genes responsible for producing OTA has increased [110,111]. Ochratoxin A biosynthesis was previously unknown. A new insight into OTA biosynthetic pathway was given through deletion of a non-ribosomal peptide synthetase gene in *A. carbonarius* [110]. A recently reported complete genome sequence of the filamentous fungus *A. westerdijkiae* illustrated the putative biosynthetic gene cluster of OTA and the genome of *A. westerdijkiae* that contains more than 50 secondary biosynthetic gene clusters where most of them were type I polyketide synthase (PKS) and non-ribosomal peptide synthase (NRPS) gene clusters [111]. Around 716 cytochrome P450 enzymes, 633 carbohydrate-active enzymes, and 377 proteases were involved in ochratoxin biosynthesis [111,112]. Furthermore, two hybrid *t1pks-nrps* gene clusters were also involved in OTA biosynthesis [112]. Patulin biosynthesis is still under investigation with omics tools [113]. More information was recently revealed relating to the genes responsible for patulin production by using different fungal species [114]. A recent study identified two strains of *Penicillium* spp. producing patulin [110]. This study provided significant information related to the molecular network of patulin biosynthesis and mechanisms of fungal host interactions specifically for *Penicillium* spp. The genome sequence of the isolates of *P. expansum* (33.52 Mb) and *P. italicum* (28.99 Mb) revealed 55 gene clusters related to secondary metabolites of which 15 genes were related to patulin biosynthesis [115]. Similarly, 15 genes were involved in patulin biosynthesis by *Aspergillus clavatus.* To date, only four genes encoding 6-methylsalicylic acid synthase, *m*-cresol hydroxylase, *m*-hydroxybenzyl alcohol hydroxylase, and isoepoxydodehydrogenase have been characterised [114]. However, the complete genome for *Aspergillus clavatus* is still not available. The explanation of the fungal genome sequence data can be achieved by using the other fungal EST database such as the NCBI GenBank database [14], *A. flavus* EST database, *A. oryzae* EST database, and the *A. oryzae* whole genome sequence. However, comparing the genome sequence may identify the differences in genome structure, significant pathogenic characters, and secondary metabolite (SM). Table 4 summarised different fungal isolates according to their genome size and their ability to produce mycotoxin.

## 4. Transcriptomics Approach

Transcriptomics is one of the most recently developed fields emerging from the genomic era [117]. After the completion of genomic studies, research has focused attention on finding the next step of gene expression and cellular functions. The genetic information stored in DNA and cannot be transcribed into proteins. Therefore, for protein synthesis, DNA is copied to RNA in a process called transcription. This step is the essential regulatory step for gene expression [118]. Thus, the transcriptomics is the study of the complete and whole set of RNA, transcript from the genome produced by genes under specific conditions from a specific tissue or cell type [56,57].

Transcriptome analysis allows researchers to understand the expression of the genome at the transcription level that provides information on the gene structure. For instance, a sequence that encodes with a direct function or an intermediate then later translated into protein; increase or decrease the production of protein (gene expression regulation), protein modifications, functions of the synthesised gene products, and evolutionary changes of the end biological processes [119,120]. Therefore, genes tend to regulate and express in different biological and physiological conditions, and accordingly, different proteins could be synthesised. For example, transcribed genes from mycotoxin infected plant cells are different from the transcribed genomes of the non-mycotoxin infected plant cell. Hence, they are essential in signaling and biochemical processes.

Transcriptomics and advanced analytical tools together play a major role in understanding complex biological systems and help to develop novel biomarkers. Thus, it has the potential ability for early stage diagnosis and in finding effective treatments in the medicinal or agricultural industry [121]. Furthermore, transcriptome analysis would further reveal the regulation network of biological processes and eventually to help in crop improvement [119,122].

### Transcriptional Profiling

Transcriptional profiling is the comprehensive study of the complete set of RNA transcripts from the genomes of a cell or tissue or an organism. Many methods have been used to study the RNA transcripts such as Northern blots, nylon membrane arrays, reverse transcriptase quantitative PCR (RT-qPCR), and the serial analysis of gene expression (SAGE). Currently, gene expression microarrays and whole transcriptome shotgun sequencing (WTSS) are the most rapid, widely used, and high-throughput tools used in transcriptome quantification [47,123,124,125]. In mycotoxin research, RT-qPCR, high-throughput microarray analysis and shotgun analysis (RNA-seq) have been extensively used [126]. Most of the transcriptomic studies on aflatoxins, ochratoxins, and patulin investigated the fungus-plant crosstalk, the abiotic factors affecting mycotoxin production and mycotoxin toxicity mechanisms. A recent study indicated that RNA sequencing data on *A. favus* infection on *Zea mays* was used to identify genes of interest involved in this cross-species network. The genes identified found to have a connection between aflatoxin production and vascular transport. Moreover, the study indicated that mycotoxin-producing fungus *A. flavus* utilised different mechanisms in response to the induction of resistance genes in *Z. mays* during the early interaction of the two organisms [127]. Transcriptional analysis of maize kernels used to identify the resistance genes with response to the aflatoxins produced by *A. flavus* and comparing with known aflatoxin defense genes was carried out by Shu et al. [128]. This study showed that genes identified during the infection of maize were expressed during the early or late stage of infection [128]. Another study used RNA sequencing and transcriptomic profiling to differentiate the gene response of *A. flavus* infected and susceptible peanut genotypes. A large number of genes was altered due to aflatoxin production. Thus, identification of the genes that are responsible for the host-pathogen crosstalk could be used in breeding resistant varieties [129]. Another study suggested that production of fungal metabolites are associated with the stress conditions. Transcriptomics study of mycotoxin-producing fungi *A. flavus* in different concentration of H_2_O_2_-supplemented media to induce oxidative stress was conducted. The results showed that isolates which produced higher levels of aflatoxin exhibited fewer differentially expressed genes in increased stress conditions. Thus, secondary metabolites including aflatoxins may be produced as a response to the oxidative stress conditions [130]. For Ochratoxin, a recent review of transcriptomic studies indicated that the transcriptomic studies carried out do not clarify the modes of action of OTA but have contributed to relevant toxicological information. Therefore, suggested to integrate the transcriptomic studies with classical toxicology studies and other omics tools to have a better understanding of the factors that contribute to OTA modes of action [131]. Table 5 summarises the studies related to transcriptomics studies on aflatoxins, ochratoxins, and patulin.

It is obvious that a considerable amount of work still needed to be carried out on transcriptional analysis of AFB_1_, OTA and PAT to extract more useful information on the biosynthesis of mycotoxin at the transcriptomics level, which opens new avenues to study and understand the biology behind these mycotoxins. This is particularly true for PAT, which we anticipate will lead to a future control strategy.

## 5. Proteomics Approach

The terms “proteome” were introduced by Marc Wilkins in 1986 [140]. Proteomics is analogous to genomics, which applies the evolving synergistic technologies of molecular biology, biochemistry, genetics, and analytical chemistry to analyse the gene products, i.e., proteins [14,141]. With the availability of genomic information and improvements in the sensitivity and throughput of analytical technology, proteomics is becoming increasingly important for many different aspects of related studies. Since proteins serve as important components of major signaling and biochemical pathways, studies at protein levels are essential to reveal molecular mechanisms to underlying in the biological processes [142]. Genes that code for enzymes essential to basic cellular functions are expressed in all cell types, whereas those with specific functions are expressed only in specific cell types. Thus, proteomics is a more complicated process than the genomics since the genome of an organism is constant while proteome differs depending on biotic and abiotic factors [14]. Due to the presence of a vast number of proteins in a cell, their analysis is a challenging task; as well as the sample preparation [143].

With the recent development of mass spectrometry (LC-ESI-MS/MS (tandem mass), MALDI-TOF (matrix-assisted laser ionisation-time of flight) mass spectrometry, protein analysis, and sequencing have become greener techniques since protein identification, and quantification was laborious and required a large amount of sample [144]. The fact that smaller amounts of material are now sufficient, faster analysis, the ability to the throughput of large numbers of samples, and robustness makes MS-based protein analysis or “proteomics” a popular and rapidly evolving area of research especially in looking at biomarkers for disease identification in medicine and agricultural research [144,145]. In this respect, the nano-scale LC-MS/MS technique has been a widely used tool in proteomics. This also enables higher sensitivity for the detection of peptides when the sample is limited [146,147]. Mass spectrometry is capable of extracting data from relatively complex mixtures of peptides. More importantly, if the complexity of the mixture is low, there is a high chance of identification of peptides. Consequently, the complexity of the sample presented for analysis can be reduced after the breakdown of the biological sample and generation of a cocktail of cells and cellular components, by the separation of proteins from the mixture. Techniques using gel electrophoresis (1D and 2D), chromatographic separations (ion exchange, size exclusion, affinity and reversed phase) can help to reduce the complexity. However, these methods cannot be used to detect the proteins due to the presence of unresolved peptides and proteins. Therefore, to analyse complex mixtures of peptides, electrophoresis or chromatographic methods are insufficient [148], and other techniques need to be employed. These include 2D gel electrophoresis which is widely used in proteomics as it simplifies the complex protein mixtures by resolving them into individual proteins or small groups of proteins [149]. The tryptic digestion of proteins produces the highest yield of peptides of optimal length for MS analysis [141,144]. Therefore, in the proteomic analysis, the cleaved peptides after the trypsin digestion are analysed by LC and data-dependent mass spectrometric methods. The peptide sequences are subsequently identified based on the obtained MS/MS data using software such as ProteinPilot against specified databases such as UniProt and National Centre for Biotechnology Information (NCBI). For example, researchers have utilised 2D gel electrophoresis with isobaric tags for relative and absolute quantitation (iTRAQ) analysis. The iTRAQ method used in quantitative proteomics by mass spectrometry techniques (tandem MS/MS) or (MALDI TOF MS/MS) to determine the number of proteins from different sources in a single experiment to identify potential biomarkers in response to fungal infection [150,151,152,153,154,155,156].

Proteomics research in mycotoxicology field not only improves our understanding of cellular behaviour by studying the different patterns of protein content but also enhances information on the effect of biotic factors and how they induce variations on the protein profile during the development of crop or the mycotoxigenic fungi. Additionally, it provides information about how the plants response to fungal infection and mycotoxin production and how they contribute to plant disease processes. A recent comparative study investigated proteome analysis of *Penicillium verrucosum* that was fermented under short wavelength light and showed stress-related proteins associated with mycotoxin biosynthesis [157]. Another study used a proteomics approach to modify cuminaldehyde thiosemicarbazone structure which induces the inhibition of aflatoxin biosynthesis and sclerotial development in *Aspergillus flavus* [158]. Such studies indicated the possible use of proteomic approaches to identify new proteins that possess fungistatic or anti-aflatoxigenic activity. Table 6 summarises the proteomics studies for aflatoxin, ochratoxin, and Patulin. It highlights the greater number of proteomics studies for aflatoxin than for ochratoxin and patulin. The use of proteomics as a novel tool in aflatoxin research has been reviewed [159]. There are many factors that influence mycotoxin biosynthesis such as fungal species, host plants, nutritional and environmental signals [160]. Nöbauer and co-authors were able to provide a comprehensive protein identification for *P. verrucosum* by using shotgun proteomics [160]. They were able to get further information about the role of adaptive changes on fungal physiology that effects secondary metabolite production [160]. Additionally, drought stress and preharvest aflatoxin contamination on the groundnuts (*Arachis hypogaea* L.) also has been reviewed recently concluding that more research is required to investigate the genes that related to resistance associated proteins [161]. Wang and co-authors used proteomic tools to analyse the immune response in cotyledons of *Arachis hypogaea* infected with *Aspergillus flavus* [162].

Proteomics approach was used to identify the biomarkers produced by *pathogenic A. flavus* as a response of oxidative stress. High, moderate and no aflatoxin producing *A. favus* were used in this study and identified more than 1000 proteins. Among them, 220 were differentially expressed [163]. Additionally, the proteome analysis of the fungus *Aspergillus carbonarius* under ochratoxin A producing conditions were investigated. The proteome analysis identified nine proteins possibly involved in diverse biological functions, two of them (acetyl glutamate kinase and TBA1_emeni tubulin alpha-1 chain) were linked to OTA production [155]. For proteomics studies on PAT, we identified four articles after researching PubMed using proteomics and PAT as keywords. Most of the studies identified were investigating the proteome changes in *Penicillium expansum* during spore germination [164] and fungal development on different media to investigate the pathogen-host interaction mechanism [165]. The discovery of proteins signals in response for mycotoxin production and was used as biomarkers, which is valuable information for the researcher to improve plant resistance and stress tolerance of host plants against fungal contamination [166].

## 6. The Current Status of Omics Studies and Future Opportunities

Despite our limited understanding of mycotoxin production, the omics approach is a vital tool in the mycotoxin field. Mycotoxin analysis has moved over ten years from targeted analysis of individual mycotoxin to untargeted metabolomics which provides insight to detect unknown metabolites. Omics tools have contributed to our understanding of mycotoxin issues especially in identifying mycotoxigenic species, information that was not accessible until recently. Additionally, it allows the identification of targeted and untargeted mycotoxins before, during, and after harvesting, highlighting possible plant-fungal interactions. Additionally, critical information is revealed on the impact of climate change on the prevalence of mycotoxin issues in society.

However, many aspects of the omics experiment are still under active development and integration of omics studies will ultimately provide accurate information about the biomarkers that relate to early-stage mycotoxin production. This task is a global challenge as it requires large scale multi omics experiments investigating the mycotoxin issue considering both biotic and abiotic factor. The development of databases internationally linked to mycotoxigenic fungi and mycotoxins would allow global integration of experimental approaches and allow comparative genomic and metabolomics studies to enable the accurate identification of mycotoxigenic strains. It would support the elucidation of components of genomes that are responsible for variation in mycotoxin production. Additionally, it would enable the identification and characterisation of plant and fungal factors that can impact mycotoxin contamination. Recognition of this important aspect has been highlighted by the research, education and economics information system (United States Department of Agriculture, USDA), which has started a project using genomic and metabolomics approaches for the detection and control of mycotoxins on corn [160]. The project is funded until 2020 and aims to develop a strategy to control mycotoxin contamination by using omics tools. It also aims to develop a DNA database to assist the scientist in identifying pathogenic strains. Similarly, EU’s Horizon 2020 programme has funded the MyToolBox project (www.mytoolbox.eu) for four-years (2016–2020). The project aims to develop new methods by using omics tool to predict potential fungal contamination of cereals at an early growth stage as well as to reduce aflatoxin contamination in EU maize through resistant plant cultivars.

The future omics studies need to provide phenotypic data considering factors such as experimental design, time, and dose responses. Integration of omics techniques must focus on investigating the pathogen-plant crosstalk, investigating the toxicity of mycotoxins on the host plant to propose mechanisms of the cell responses, which will direct efforts to mitigate the mycotoxin issue.

By combining omics technologies, functional genomics, transcriptomics, proteomics, and metabolomics along with bioinformatics is needed to provide extensive information about the biotic and abiotic factors that contribute to the global mycotoxin issue. With genomics, the comprehensive understanding based on the gene and molecular level will assist in the search to develop novel strategies to control mycotoxin contamination by identifying targets for inhibiting fungal growth or toxin production. Proteomics and transcriptomic information can contribute to developing online screening methods which will help farmers to predict fungal contamination in the early stages of development. Additionally, omics technologies contribute to our understanding about pathogen fungal crosstalk that could accelerate the development of plant breeding through gene insertion technologies for enhancing host plant resistance, preventing or reducing mycotoxin contamination in pre- and post-harvest crops.

## Figures and Tables

**Table 1:** Polyketide derived mycotoxins.

Mycotoxin	Structure	Fungal Species	IARC Classification	Ref.
Aflatoxin B_1_	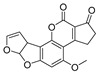	*Aspergillus flavus*, *Aspergillus parasiticus*, *Aspergillus bombycis*, *Aspergillus A. coracles*, *Aspergillus nomius*, *Aspergillus pseudotamari*	Group A carcinogen	[1,13,15,16,17]
Ochratoxin A	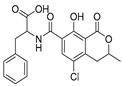	*Aspergillus alliaceus*, *Aspergillus melleus*, *Aspergillus cabonarius*, *Aspergillus glaucus*, *Aspergillus niger*, *Penicillium viridicatum*	Group 2B possible human carcinogen	[13,18,26]
Patulin	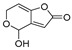	*Penicillium expansum*, *Penicillium patulum*, *Penicillium crustosum*	Group 3	[19,25]

**Table 2:** Metabolomics analysis applications and detection performance criteria for targeted and untargeted mycotoxin detection.

Toxin	Crops	Detection Techniques (Targeted or Non-Targeted)	LoD *	LoQ *	Ref.
AFB_1_, G_1_	Peanuts, corn, soy beans	**Targeted and Non-targeted** HPLC-ESI-MS-qTOF, ESI^+^ UHPLC-ESI-MS/MS, sSRM	0.1–0.3 µg/kg	0.2–0.9 µg/kg	[50]
AFB_1_, B_2_, G_1_, G_2_, M_1_, OTA	Feed and feed raw materials (silage, maize, wheat, wheat by-products, barley, soy beans, sunflower seeds)	**Targeted and Non-targeted** LC-ESI-MS/MS (QTRAP) ESI^+^, ESI^−^, sSRM	n/a	n/a	[72]
AFB_1_, B_2,_ G_1_, G_2_, M_1_, M_2_, OTA, OTB, Patulin	Almonds, hazelnuts, peanuts, pistachio	**Targeted** UHPLC-ESI-MS/MS (qQq), ESI^+^, ESI^−^, sSRM	n/a	AFB_1_ 3.0 µg/kg AFB_2_ 10.0 µg/kg AFG_1_ 10.0 µg/kg AFG_2_ 8.2 µg/kg AFM_1_ 7.9 µg/kg OTA 15.0 µg/kg OTB 9.9 µg/kg PAT n/a	[53]
AFB_1_, B_2_, G_1_, G_2_, OTA	Barley	**Targeted** GC-MS/MS (qQq), EI, derivatizied, LC-ESI-MS/MS (QTRAP), ESI	AFs 2.0 ng/kg OTA 2.0 ng/kg	AFs 3.5 ng/kg OTA 3.5 ng/kg	[58]
AFB_1_, B_2_, G_1_, G_2_	Rice, sorghum	**Targeted** LC-ESI-MS/MS or UHPLC-ESI-MS/MS (tandem quadrupole), ESI^+^, sSRM	0.1–1.0 µg/kg	0.28–0.9 µg/kg	[73]
AFB_1_, B_2_, G_1_, G_2_, OTA	Wheat, corn and rice cereals	**Targeted** UHPLC-ESI-MS/MS (tandem quadrupole) ESI^+^, sSRM	0.1–5.0 µg/kg, (AFB_1_ 0.03 µg/kg)	0.1–25.0 µg/kg	[74]
AFB_1_, B_2_, G_1_, G_2_, M_1_, OTA	Various foods and feed (24 types of corn feeds, peanut butter)	**Targeted** UHPLC-ESI-MS/MS (qQq tandem) ESI^+^, ESI^−^, sSRM	AFs 0.003 µg/kg AFG_2_ 0.006 µg/kg OTA 0.064 µg/kg	AFs 0.01 µg/kg AFG_2_ 0.02 µg/kg OTA 0.21 µg/kg	[75]
AFB_1_, B_2_, G_1_, G_2_, OTA	Maize	**Targeted** LC-ESI-MS/MS (QTRAP qQq) ESI^+^, ESI^−^, sSRM	AFB_1_ 0.6 µg/kg AFB_2_ 0.3 µg/kg AFG_1_ 0.4 µg/kg AFG_2_ 0.8 µg/kg OTA 0.6 µg/kg	n/a	[76]
AFB_1_, B_2_, G_1_, G_2_, OTA	Barley based breakfast cereals, maize, peanuts	**Targeted** UHPLC-ESI-MS/MS (QTRAP qQq) ESI^+^ ESI^−^ (in single run), sSRM	AFs 0.05 µg/kg OTA 0.1 µg/kg	AFs 0.1 µg/kg OTA 0.25 µg/kg	[77]
AFB_1_, B_2_, G_1_, G_2_, OTA	Durum wheat, corn flakes, maize and maize crackers	**Targeted** LC-ESI-MS/MS (QTRAP qQq) ESI^+^ ESI^−^, sSRM	n/a	AFs 1.0 µg/kg OTA 1.0 µg/mg	[78]
AFB_1_, B_2_, G_1_, G_2_, OTA	Muesli, wheat flakes, oats, raisins, sultanas, whey powder, hazelnuts, whole meal bread	**Targeted** LC-ESI-MS/MS (tandem quadrupole) ESI^+^, sSRM	AFB_1_ 0.05 ng/g AFB_2_ 0.03 ng/g AFG_1_ 0.03 ng/g AFG_2_ 0.03 ng/g OTA 0.03 ng/g	AFB_1_ 0.1 ng/g AFB_2_ 0.05 ng/g AFG_1_ 0.05 ng/g AFG_2_ 0.05 ng/g OTA 0.4 ng/g	[79]
AFB_1_, B_2_, G_1_, G_2_, OTA	Barley, corn, corn gluten, infant cereals, oat, rice, rye, wheat	**Targeted** LC-ESI-MS/MS (QTRAP qQq tandem mass) ESI^+^, ESI^−^, sSRM	n/a	AFs 1.0–10.0 µg/kg OTA 0.5–2.5 µg/kg	[80]
AFB_1_, B_2_, G_1_, G_2_, OTA	Maize, rice, wheat	**Targeted** LC-ESI-MS/MS (qQq tandem) ESI^+^, sSRM	AFB_1_ 0.12–0.21 g/kg AFB_2_ 0.06–0.7 µg/kg AFG_1_ 0.07–2.3 µg/kg AFG_2_ 0.11–2.2 µg/kg OTA 0.18–3.2 µg/kg	AFB_1_ 0.12–0.21 µg/kg AFB_2_ 0.06–0.7 µg/kg AFG_1_ 0.07–2.3 µg/kg AFG_2_ 0.11–2.2 µg/kg OTA 0.18–3.2 µg/kg	[81]
AFs, OTA	Black pepper, infant food (apple baby food), paprika, sunflower seed, wheat flour	**Targeted** UHPLC-ESI-MS/MS (QTRAP tandem) ESI^+^, ESI^−^, sSRM, Non-targeted UHPLC-ESI-HRMS (TOF) ESI^+^ ESI^−^	n/a	n/a	[82]
AFB_1_, B_2_, G_1_, G_2_, OTA, OTB, OTC, Patulin	Maize, wheat	**Targeted** HPLC-ESI-MS/MS (QTRAP qQq), ESI^+^, ESI^−^, sSRM	0.03–220 µg/kg	n/a	[83]
AFB_1_, B_2,_ G_1_, G_2_, M_1_, OTA, Patulin	Apple puree, green pepper, hazelnut, maize	**Targeted** UHPLC-ESI-MS/MS (QTRAP) ESI^+^, ESI^−^, sSRM	AFB_1_ 0.6 µg/kg AFB_2_ 0.6 µg/kg AFG_1_ 1.2 µg/kg AFG_2_ 2.3 µg/kg AFM_1_ 0.6 µg/kg OTA 1.2 µg/kg PAT 35.9 µg/kg	AFB_1_ 1.9 µg/kg AFB_2_ 4.0 µg/kg AFG_1_ 7.6 µg/kg AFG_2_ 8.7 µg/kg AFM_1_ 2.1 µg/kg OTA 3.7 µg/kg PAT 119.7 µg/kg	[65]
AFB_1_, B_2_, G_1_, G_2_, OTA	Barley	**Targeted** UHPLC-HRMS (Orbitrap) Heated EPI (HEPI), HEPI^+^, HEPI^−^	n/a	n/a	[84]
OTA	Barley	**Targeted** UHPLC-FTHRMS HEPI, HEPI^+^, HEPI^−^	n/a	n/a	[85]
AFB_1_, B_2_, G_1_, G_2_, OTA	Black radish, Ginkgo biloba, garlic, soy	**Targeted** UHPLC-ESI-MS/MS (qQq), ESI^+^, sSRM	AFs 6.0 ng/g OTA 1.0 ng/g	AFs 2.0 ng/g OTA 0.3 ng/g	[86]
AFB_1_, B_2_, G_1_, G_2_, M_1_, OTA, OTB	Maize, groundnut, sorghum, millet, rice, wheat, soy, dried fruits, infant foods, other processed food, animal feed	**Targeted** HPLC-ESI-MS/MS (QTRAP) ESI^+^, ESI^−^, sSRM	AFB_1_ 3.0 µg/kg AFB_2_ 6.0 µg/kg AFG_1_ 8.0 µg/kg AFG_2_ 8.0 µg/kg AFM_1_ 4.0 µg/kg OTA, OTB 5.0 µg/kg	n/a	[87]
AFB_1_, B_2_, G_1_, G_2_, M_1_, OTA	Breakfast cereals (maize, wheat, rice, multigrain, chocolate)	**Targeted** HPLC-fluorescence detector-EI-MS/MS, sSRM	AFB_1_ 0.003 µg/kg AFB_2_ 0.001 µg/kg AFG_1_ 0.006 µg/kg AFG_2_ n/a AFM_1_ 0.011 µg/kg OTA 0.006 µg/kg	AFB_1_ 0.009 µg/kg AFB_2_ 0.004 µg/kg AFG_1_ 0.018 µg/kg AFG_2_ n/a AFM_1_ 0.032 µg/kg OTA 0.019 µg/kg	[88]
OTA	Wheat flour, coffee, spices, wine, beer	**Targeted** HPLC-MS/MS (ion trap), (1) ESI^+^ (2) APCI, sSRM	0.5 µg/kg	1.4 µg/kg	[89]
AFB_1_, B_2_, G_1_, G_2_	Peanut, peanut butter, spices, figs	**Targeted** LC-APCI-MS/MS (qQq), APCI^+^, sSRM, targeted	0.1 µg/kg	n/a	[90]
Patulin	Wheat, rice, spelt, oat, soy, tapioca based cereals (cassava), pasta, infant food	**Targeted** GC-MS/MS (qQq), electron impact ion source (EI), SRM, derivatizied, targeted	n/a	5–10 µg/kg	[56]

AFs—aflatoxins, AFB_1_—aflatoxin B_1_, AFB_2_—aflatoxin B_2_, AFG_1_—aflatoxin G_1_, AFG_2_—aflatoxin G_2_, AFM_1_—aflatoxin M_1_, APCI—atmospheric pressure chemical ionization; EI—electron impact ionization; ESI—electrospray ionization; GC—gas chromatography; HEPI—heated electron spray ionization; HRMS—high resolution mass spectrometry; LC—liquid chromatography; qQq—triple quadrupole; QTOF—quadrupole time of flight; sSRM—scheduled selected reaction monitoring; TOF—time of flight; UHPLC—ultra-high pressure liquid chromatography; n/a—not available) LoD—limit of detection, LoQ—Limit of quantificatin.

**Table 3:** Genomic tools used in mycotoxin research.

Genomic Tools	Mycotoxins	Crops	Comments	Ref.
Ion Torrent Personal Genome Machine (PGM)	Aflatoxins	—	Whole genome sequencing	[99]
Microarray analysis, quantitative reverse transcription-PCR (qRT-PCR)	Aflatoxins	—	Aflatoxin biosynthesis	[100]
Microarray analysis	Aflatoxins	—	Whole genome sequencing	[101]
Microarray analysis	Aflatoxins	—	Gene expression profiles	[102]
Whole genome sequencing		—	Identify genes differentially expressed in wild-type veA and veA mutant strains that could be involved in aflatoxin production.	[92]
RT-PCR and reverse-transcription PCR		Peanuts	Develop a screening method	[96]
PCR and LAMP-based group specific		Rice, nuts, raisins, dried figs	Develop a screening method to detect several aflatoxin producing species in a single analysis	[103]
Microarray	Aflatoxins, ochratoxin A	Wheat grain	Rapid detection for mycotoxins	[104]

**Table 4:** Mycotoxigenic strains and their genome sizes.

Fungal Strains	Genomic Size (Mbp *)	Mycotoxin	Mycotoxigenic	Ref.
*LOAM00000000 flavus*	36.0	Aflatoxin	Yes	[108]
*LIZI00000000 flavus*	36.4	Aflatoxin	Yes	
*LIZJ00000000 flavus*	36.3	Aflatoxin	Yes	
*LOAK00000000 flavus*	35.9	Aflatoxin	Yes	
*LOAL00000000 flavus*	35.8	Aflatoxin	Yes	
*LOAP00000000 parasiticus*	30.1	Aflatoxin	Yes	
*NRRL 13137 nominus*	36.1	Aflatoxin	Yes	[99]
*Aspergillus korhogoensis*	N/a	Aflatoxin	Yes	[116]
*Aspergillus westerdijkiae*	36.1	Ochratoxin A	Yes	[111]
*Aspergillus carbonarius*	36	Ochratoxin A	Yes	[110]
*Penicillium expansum*	*33.52*	Patulin	Yes	[115]
*Penicillium italicum*	28.99	Patulin	Yes	[115]

* Mbp-mega base pairs.

**Table 5:** Transcriptomics studies for aflatoxin, ochratoxin, and patulin.

Mycotoxin	Studies	Outcomes	Ref.
Aflatoxin B_1_	Identification of essential transcription factors for adequate DNA damage response after benzo (*a*) pyrene and aflatoxin B_1_ exposure by combining transcriptomics with functional genomics.	Transcriptomics and functional genomics tools used to investigate the genotoxicity of aflatoxin B_1_.	[132]
	Aflatoxin B_1_ induces persistent epigenomic effects in primary human hepatocytes associated with hepatocellular carcinoma.	Transcriptomics and epigenome studies used to understand the mechanisms of hepatocellular carcinoma development.	[8]
	Quercetin tests negative for genotoxicity in transcriptome analyses of liver and small intestine of mice.	Genotoxicity related pathways in mice liver and small intestine.	[133]
	Combined cytotoxicity of aflatoxin B1 and deoxynivalenol to hepatoma HepG2/C3A cells.	Different cytotoxicity pathways and their apoptotic process might be the mechanism of the synergistic cytotoxicity of HepG2/C3A carcinoma cells.	[134]
	Integrated analysis of transcriptomics and metabolomics profiles in aflatoxin B1-induced hepatotoxicity in rat.	Gluconeogenesis, lipid metabolism disorder, and induced hepatotoxicity affect majorly after the acute AFB1 exposure.	[122]
	Identification of early target genes of aflatoxin B1 in human hepatocytes, inter-individual variability and comparison with other genotoxic compounds.	Gene subset from AFB1 induced human hepatocytes identified several genes which are potential biomarkers of genotoxic compounds.	[135]
Aflatoxins	Use of functional genomics to assess the climate change impact on *Aspergillus flavus* and aflatoxin production.	Global temperature, water availability and rising CO_2_ levels affect the expression of the aflatoxin biosynthetic regulatory gene *aflR*.	[109]
Ochratoxin A	Different toxicity mechanisms for citrinin and ochratoxin A revealed by transcriptomic analysis in yeast.	OTA deregulates developmental genes.	[136]
	Disruption of liver development and coagulation pathway by ochratoxin A in embryonic zebrafish.	OTA exposure led to a deficiency of coagulation factors.	[137]
	Transcriptomic alterations induced by OTA in rat and human renal proximal tubular in vitro models and comparison to rat in vivo model.	The study provided a non-genotoxic mechanism of OTA-induced carcinogenicity.	[138]
Patulin	Transcriptomic responses of the basidiomycete *Sporobolomyces* sp. to the mycotoxin patulin.	Exposure to PAT directed the changes in gene expression in *Sporobolomyces* sp. This finding may lead to develop a bio-detoxification process.	[139]

**Table 6:** Proteomics studies for aflatoxin, ochratoxin and patulin.

Mycotoxin	Fungal Strains	Study	Analysis Techniques	Outcome	Ref.
Aflatoxin B_1_	*Aspergillus flavus*	Proteomic analysis reveals an aflatoxin-triggered immune response in cotyledons of *Arachis hypogaea* infected with *Aspergillus flavus.*	2-D gel electrophoresis and MALDI-TOF/TOF mass spectrometer.	Three grades of the immune response in *A. hypogaea* during infection with toxigenic *A. flavus* were identified. PAMP-triggered immunity, effector-triggered immunity and metabolite-triggered immunity.	[162]
	*Aspergillus flavus*	Comparative leaf proteomics of drought-tolerant and-susceptible peanut in response to water stress.	2-D gel electrophoresis and MALDI-TOF/TOF mass spectrometer.	42 unique proteins showed interactions in the tolerant cultivar.	[167]
	*Aspergillus flavus*	Insight into the global regulation of laeA in *Aspergillus flavus* based on proteomic profiling	Protein extraction, trypsin digestion, TMT-labelling and HPLC fractionation and LC-MS/MS	laeA gene affects cell morphology and contributes to the production of aflatoxin production.	[168]
	*Aspergillus flavus*	Proteome analysis of *A. flavus* isolate-specific responses to oxidative stress in relationship to aflatoxin production capability.	Protein digestion and iTRAQ * labelling	1173 proteins were identified, and 220 were differentially expressed.	[163]
Ochratoxin A	*Aspergillus carbonarius*	Proteome analysis of the fungus *Aspergillus carbonarius* under ochratoxin A producing conditions.	2-D gel electrophoresis and MALDI-TOF/TOF mass spectrometer.	Nine differential proteins were identified by MALDI-MS/MS and MASCOT. Identified proteins were involved in regulation, amino acid metabolism, oxidative stress and sporulation. A protein with 126.5 fold higher abundance in high OTA-producing strain showed homology with CipC.	[155]
	*Arabidopsis thaliana*	iTRAQ mitoproteome Analysis reveals mechanisms of programmed cell death in *Arabidopsis thaliana* induced by ochratoxin A	iTRAQ * Analysis	The study investigated the toxicity mechanism of OTA on the host plant; their results indicated that OTA induced PCD in *A. thaliana*. 42 and 43 proteins were identified within 8 and 24 h. those proteins were mainly involved in perturbation of the mitochondrial electron transport chain, interfering with ATP synthesis and inducing PCD	[169]
Patulin	*Penicillium expansum*	Identification of differentially expressed genes involved in spore germination of *Penicillium expansum* by comparative transcriptome and proteome approaches.	RNA-seq (RNA sequencing) and iTRAQ * (isobaric tags for relative and absolute quantitation) approaches.	A total of 3026 differentially expressed genes, 77 differentially expressed predicted transcription factors and 489 differentially expressed proteins identified. Posttranscriptional regulation and modification serve essential roles in the management of fungal germination.	[164]

MALDI—matrix-assisted laser desorption/ionization, TOF/TOF—time-of-flight, TMT—Tandem Mass Tag, iTRAQ—isobaric tags for relative and absolute quantitation.
